# Endobronchial tuberculosis with unusual linear ulceration from tracheal to right upper lobar bronchi

**DOI:** 10.1002/rcr2.901

**Published:** 2022-01-10

**Authors:** Hung‐Yi Lin, Jih‐Chin Lee

**Affiliations:** ^1^ Division of Pulmonary and Critical Care Tri‐Service General Hospital Taipei Taiwan; ^2^ Department of Otolaryngology‐Head and Neck Surgery Tri‐Service General Hospital Taipei Taiwan

**Keywords:** bronchoscopy, endobronchial, tuberculosis

## Abstract

The complications of endobronchial tuberculosis (TB) include bronchostenosis and fistula. We highly recommend that a TB test be included in the diagnosis of endobronchial lesions.

The complications of endobronchial tuberculosis (TB) include bronchostenosis and fistula. We highly recommend that a TB test be included in the diagnosis of endobronchial lesions.

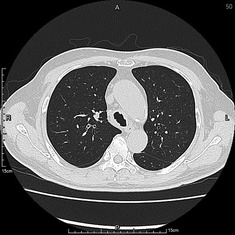

## CLINICAL IMAGE

**FIGURE 1:**
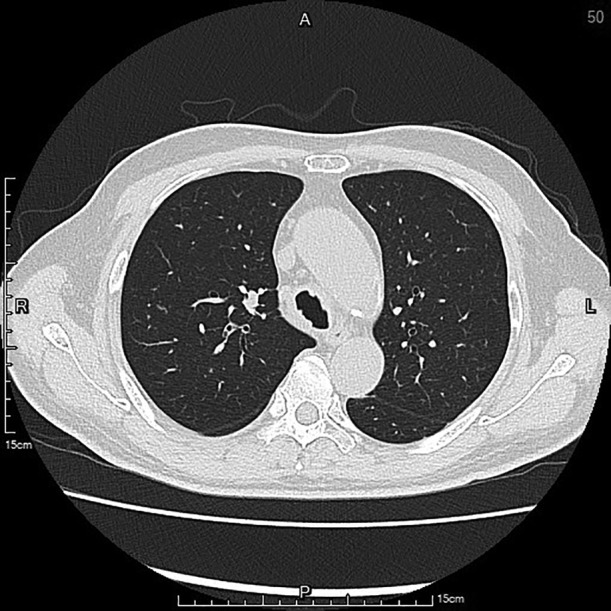
The tracheal ulceration

**FIGURE 2:**
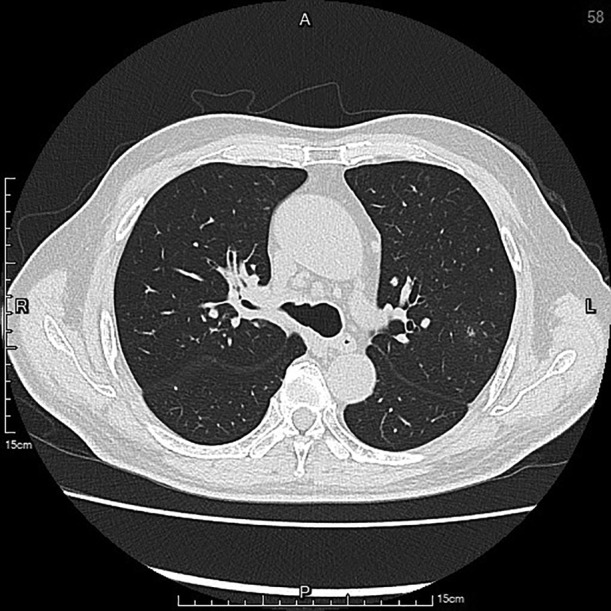
The narrowed right upper bronchi

**FIGURE 3:**
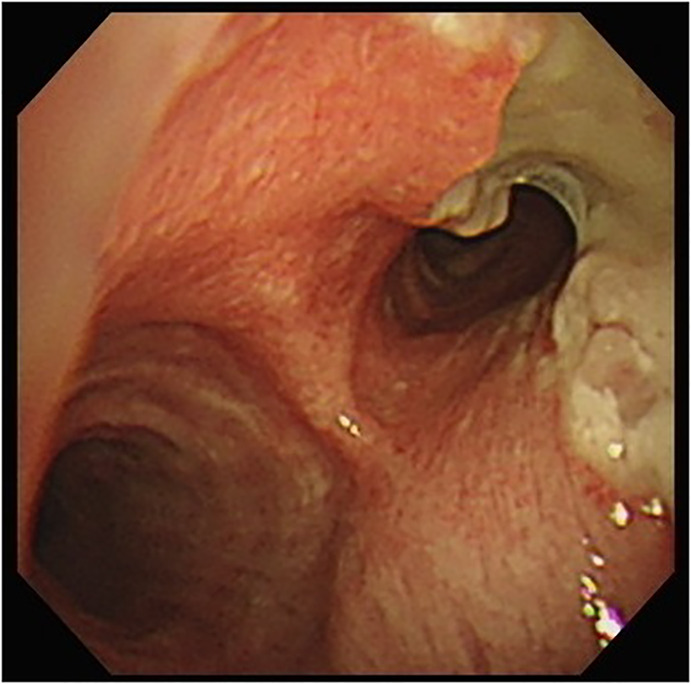
Endoscopy shows the tracheal ulceration lesion and extended right main bronchus

**FIGURE 4:**
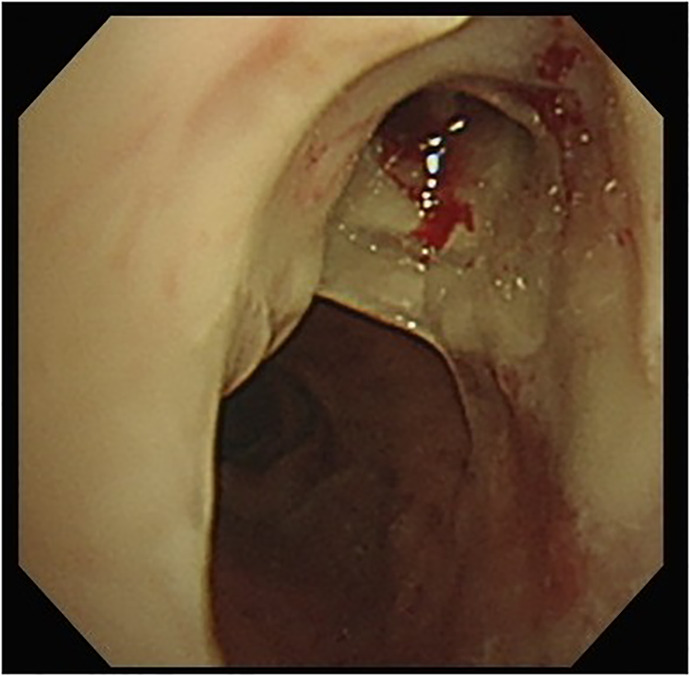
Clotting of the right upper bronchi with mucus

A 67‐year‐old hypertensive, immunocompetent male complained of cough, sore throat and hoarseness for 2 months. Computed tomography of chest showed tracheal ulceration (Figure 1), narrowed right upper bronchus and multiple bilateral small nodules (Figure 2), but no cavitation. Laryngoscopy showed ulceration below the subglottic area. Fibreoptic bronchoscopy showed linear ulceration with white secretions in the trachea (Figure 3) to the right upper lobe bronchi (Figure 4). The remainder of the bronchi appeared normal. Bronchoalveolar lavage samples were positive on acid‐fast bacilli stain and *Mycobacterium tuberculosis* DNA complex polymerase chain reaction, with tuberculosis (TB) confirmed on mycobacterial culture. Sputum samples were smear‐positive for acid‐fast bacilli. The patient had no history of TB contacts, prior tuberculous infection and no additional symptoms of weight loss, fever or haemoptysis. HIV testing was negative. Intermediate rates of TB (33.2 cases per 100,000 inhabitants) occur in Taiwan; thus, TB was highly suspected as a cause of lung infection. The patient was started on standard anti‐tuberculous treatment with isoniazid, rifampicin, ethambutol and pyrazinamide. Timely anti‐tuberculous treatment can eradicate or minimize TB transmission and decrease long‐term complications of endobronchial TB, such as bronchial stenosis. In conclusion, we highly recommend that testing for TB be included in the investigation of endobronchial lesions.

## CONFLICT OF INTEREST

None declared.

## AUTHOR CONTRIBUTION

Hung‐Yi Lin was involved in investigation, writing—original draft, writing–review and editing and final approval of the manuscript. Jih‐Chin Lee revised the manuscript. Both authors approved the manuscript.

## ETHICS STATEMENT

The authors declare that appropriate written informed consent was obtained for the publication of this manuscript and accompanying images.

## Data Availability

The data that support the findings of this study are available from the corresponding author upon reasonable request.

